# Book Reviews

**Published:** 1898-05

**Authors:** 


### BOOK REVIEWS.

ANNUAL AND ANALYTICAL CYCLOPEDIA OF PRACTICAL MEDICINE. By Charles E. de M. Sajous, M.D., and One Hundred Associate Editors, assisted by Corresponding Editors and Collaborators. Volume I. The F. A. Davis Company, Publishers, Philadelphia.

This work, which is the successor to the *Annual of the Universal Medical Sciences,* is intended to correct some of the deficiences in the latter and to supply the practitioner with a Cyclopedia covering the entire field of Medicine. "Instead of presenting the excerpts from the year's literature arranged under a general head as before, each disease (including its subdivisions, etiology, pathology, treatment, etc.) is described *in extenso,* and the new features that the year has brought forth are inserted in their respective places in the text. In this manner the reader is saved [much] the fatiguing study. . . The work, when completed, will present all the general diseases described in text-books on practical subjects, medicine, surgery, therapeutics, obstetrics, etc., and, inserted in their logical order in the text, are the progressive features of value presented during the last decade."

There will be six volumes in the completed work. The first volume includes a compact, but full, discussion (in alphabetical order) of subjects from "Abdominal Injuries" to "Bright's Disease," embracing, therefore, such important subjects as *abdomen, abortion, abscess, acne, acromegaly, Addison's disease, albuminuria, alcoholism, anemia, aneurism, animal extracts, aphasia, appendicitis, asthma, Bright's disease, etc.* Brief abstracts are given of the most important articles bearing upon each subject. The volume gives evidence of much labor in its preparation and of an earnest effort on the part of editor and collaborators to place before the practitioner a valuable general reference book in medicine which will be an ever-present help in time of need.

ATLAS OF CLINICAL INVESTIGATION. With an Epitome of Clinical Diagnosis and of Special Pathology and Treatment of Internal Diseases. By Dr. Christfried Jakob. Edited by Augustus A. Eshner, M.D., Philadelphia. W. B. Saunders, Publisher, Philadelphia.

As is truly stated in the translator's note, "The beauty, the clearness, and the accuracy of the illustrations . . . seemed to be a sufficient justification for adapting the work to the needs of American medical students and practitioners." The general scope of the work may be divided into two parts, the first treating of the microscopic appearances and chemical reactions of the blood, the stomach contents and the secretions and excretions of the body, the second part of the physical signs as found by the various methods of investigation. In conclusion, 242 pages of text are devoted to a concise résumé of physical diagnosis.

If the work contained no more than the first twenty-two plates, it would be worthy of a prominent place in the library of every physician who avails himself of microscopical and chemical aids in diagnosis. The plates, especially those on urinary examinations, are the best we have yet seen, and hardly need the accompanying text to elucidate them.

The remaining forty-six plates are beautifully executed, and clearly illustrate the physical signs of as many pathological conditions. Each one illustrates an actual case in the author's experience, and is accompanied by a short history, the results of the physical examination, the diagnosis and treatment, and in many cases the results of the post-mortem examination.

The work is a valuable one and should meet with hearty approval. F. S. B.

---

THE SURGICAL COMPLICATIONS AND SEQUELS OF TYPHOID FEVER. By W. W. Keen, M.D., LL.D., Professor of Surgery in the Jefferson Medical College, Philadelphia Published by W. B. Saunders, Philadelphia. Price $3.00 net.

This elaborate work consists of a book of three hundred and eighty-six pages, containing in addition to the observations of the writer and his assistants since 1876, his Toner Lecture, which was printed by the Smithsonian Institute at that time.

The first portion of the book is devoted to a consideration of the pathology of the complications and sequels of typhoid fever, with especial reference to the viability and diffusion of typhoid bacillus. Chapter III. is devoted to gangrene, which occurs with this disease. Other chapters enter upon the consideration of the various manifestations of the disease in the various tissues throughout the body. One of the most interesting chapters is the one on intestinal perforation, with the results of treatment from a surgical standpoint, when the perforation is recognized early and when late. In speaking of the time when an operation should be performed, he says: "The earliest moment at which the operation can be done after the immediate shock of the perforation, provided, of course, there has been any, as is sometimes not the case, the better it will be for the patient." This is a very comprehensive book and treats of this subject in a scientific and interesting style. The wide diffusion of the typhoid bacillus is especially interesting and its possible active part which it plays in the multitude of complications of this disease. E. C. D.

---

DAY-DREAMS OF A DOCTOR. By C. Barlow, M.D. Pp. 251. The Peter Paul Book Company, Buffalo, New York.

The author of this book tell us that he took his cue from the remark of Dr. Wier Mitchell, "I think there remains to be written the simple, honest, dutiful story of an intelligent, thoughtful every-day doctor, such as will pleasantly and fitly open to laymen some true conception of the life he leads—its cares, its trials, its influences on himself and others, and its varied rewards"; and Dr. Barlow believes he has presented a "fair conception of the real life of the plain, hard-working, every-day doctor."

A reading of the volume satisfies one that the writer has very well sustained his purpose. His descriptions are exceedingly real, and many of the representative incidents which he chooses to describe can be more or less duplicated in the experiences of every "busy practitioner." The writer believes in the female physician, one of whom comes in for due attention, and the male representative of the general practitioner marries her in the end; so it appears that the simple annals of busy, hard-worked "every-day doctors" are not without some romance. The chapter on "Doctor Crook" is as felicitous as it is lifelike. The author writes in a natural and easy style, which adds much to the interest of the volume.

---

AN AMERICAN TEXT-BOOK OF GENITO-URINARY DISEASES, SYPHILIS, AND DISEASES OF THE SKIN. Edited by L. Bolton Bangs, M.D., Consulting Surgeon to St. Luke's Hospital and the City Hospital, New York, etc.; and W. A. Hardaway, A.M., M.D., Professor of Diseases of the Skin and Syphilis in the Missouri Medical College, St. Louis. Illustrated with 300 engravings and 20 full-page colored plates. W. B. Saunders, 925 Walnut St., Philadelphia. Price, cloth, $7.00; half morocco, $8.00.

This is a "collaborated" book of about twelve hundred pages. The writers of the various parts are mostly well known authorities, but there are some new ones. Nearly every medical center in the country seems represented, as is Canada. The diseases coming under the title are very fully discussed, and the subjects appear thoroughly covered. Individual views crop out here and there, but quotations from the general literature abound. The colored plates are not the best. The cuts are better. One should be able to get along in the specialties treated with this book alone.

M. B. H.

---

A COMPENDIUM OF INSANITY. By John B. Chapin, M.D., LL.D. Published by W. B. Saunders, Philadelphia. Price $1.25 net.

This little book may truly be called a compendium, for it contains in a condensed form the essentials of diagnosis and treatment of the various forms of insanity. It is written in an extremely simple style and arranged so as to prove unusually interesting, not only to the physician but the lawyer who cannot devote considerable time to the more technical books on insanity. It is an attractive book in appearance and the arrangement of the various subjects handled is such as to make it well worth a careful reading.

